# Canine SOD1 harboring E40K or T18S mutations promotes protein aggregation without reducing the global structural stability

**DOI:** 10.7717/peerj.9512

**Published:** 2020-07-15

**Authors:** Shintaro Kimura, Yuji O. Kamatari, Yukina Kuwahara, Hideaki Hara, Osamu Yamato, Sadatoshi Maeda, Hiroaki Kamishina, Ryo Honda

**Affiliations:** 1 The United Graduate School of Veterinary Sciences, Gifu University, Gifu, Japan; 2 Life Science Research Center, Gifu University, Gifu, Japan; 3 Joint Department of Veterinary Medicine, Faculty of Applied Biological Sciences, Gifu University, Gifu, Japan; 4 Center for Highly Advanced Integration of Nano and Life Sciences, Gifu University, Gifu, Japan; 5 Molecular Pharmacology, Department of Biofunctional Evaluation, Gifu Pharmaceutical University, Gifu, Japan; 6 Laboratory of Veterinary Clinical Pathology, Joint Faculty of Veterinary Medicine, Kagoshima University, Kagoshima, Japan; 7 The United Graduate School of Drug Discovery and Medical Information Sciences, Gifu University, Gifu, Japan

**Keywords:** Aggregation, ALS, Degenerative myelopathy, SOD1 E40K mutation, SOD1 T18S mutation

## Abstract

Amyotrophic lateral sclerosis (ALS) is a progressive and fatal neurodegenerative disease associated with aggregation of superoxide dismutase 1 (SOD1) protein. More than 160 mutations in human SOD1 have been identified in familial ALS and extensively characterized in previous studies. Here, we investigated the effects of T18S and E40K mutations on protein aggregation of canine SOD1. These two mutations are exclusively found in canine degenerative myelopathy (an ALS-like neurodegenerative disease in dogs), whose phenotype is unknown at the level of protein folding. Interestingly, the T18S and E40K mutations did not alter far-UV CD spectrum, enzymatic activity, or global structural stability of canine SOD1. However, thioflavin-T assay and transmission electron microscopy analysis revealed that these mutations promote formation of fibrous aggregates, in particular in the Cu^2+^/Zn^2+^-unbound state. These evidence suggested that the T18S and E40K mutations promote protein aggregation through a unique mechanism, possibly involving destabilization of the local structure, reduction of net negative charge, or production of disulfide-linked oligomers.

## Introduction

**Figure 1:**
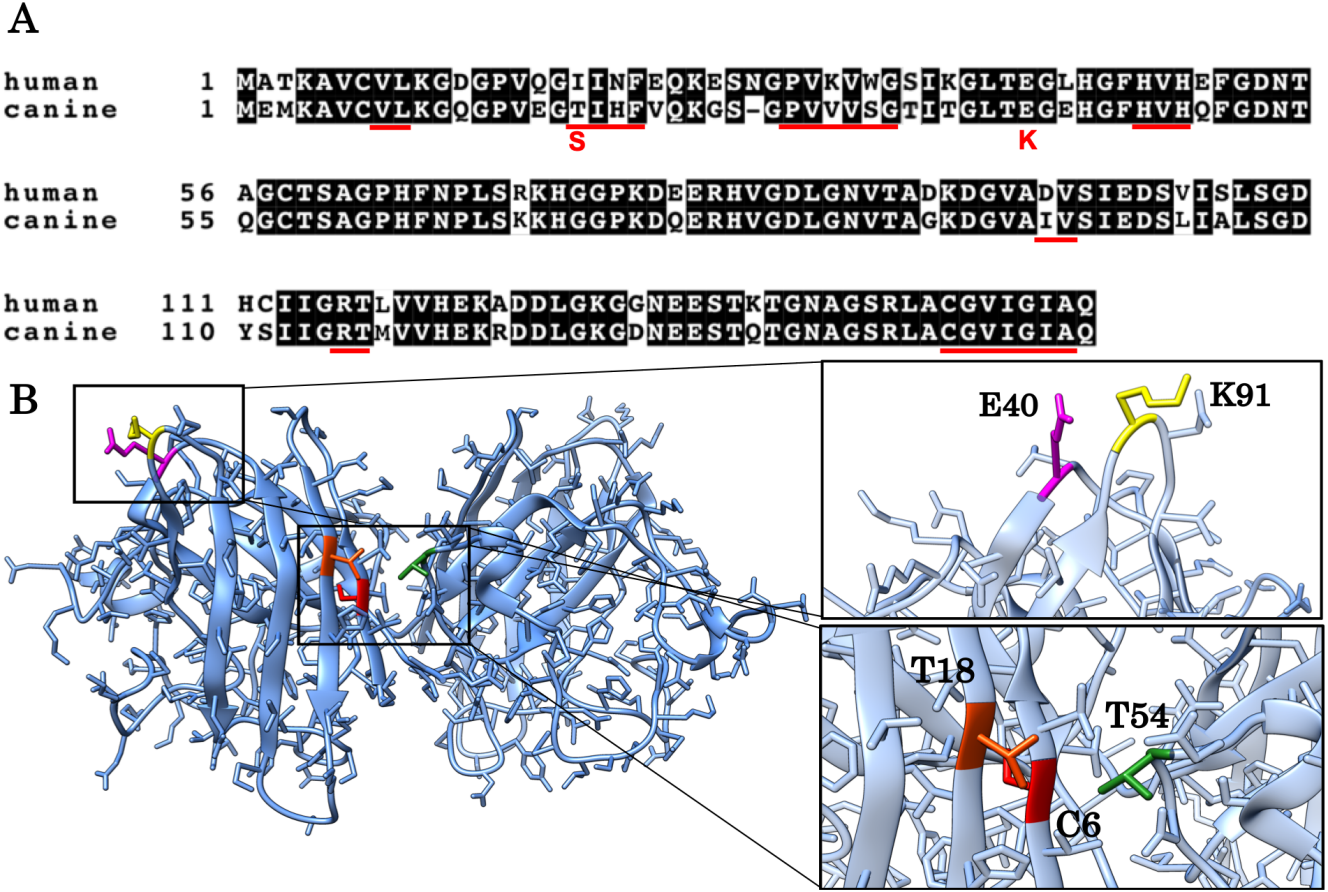
Alignment of the human and canine SOD1 amino acid sequences and a 3D homology model of canine SOD1 protein. (A) Alignment of the human and canine SOD1 amino acid sequences. Residues identical between human and canine SOD1 are presented on shaded background. The red underlines show aggregation promoting residues predicted by Zyggregator. (B) A 3D homology model of canine SOD1. The model was built based on human SOD1 (Protein Data Bank code: 3ECU) using the SWISS-MODEL web server. The T18 (orange red) and T54 residues (green) form hydrophobic interaction on the dimer interface. The C6 residue is presented in red color. The E40 (magenta) and K91 (yellow) residues form the salt bridge.

Amyotrophic lateral sclerosis (ALS) is a progressive and fatal neurodegenerative disease (Kiernan et al., 2011; Turner & Talbot, 2008; Rosen et al., 1993). More than 160 mutations in the superoxide dismutase 1 (SOD1) gene have been found in familial ALS (Dion, Daoud & Rouleau, 2009; Andersen & Al-Chalabi, 2011; Hayward et al., 1998). The SOD1 gene encodes a 153-amino acid residue protein which catalyzes the dismutation of superoxide radicals. The SOD1 protein is a stable homodimer, where each subunit is composed of eight *β*-strands with copper and zinc ions (Fig. 1). Many of the SOD1 mutations promote misfolding and aggregation of SOD1 proteins (Bruijn, Miller & Cleveland, 2004) and inhibit various cellular activities leading to neurodegeneration (Cleveland & Rothstein, 2001). Effects of several ALS-associated mutations have been extensively characterized in previous studies and suggested to destabilize the native dimeric structure (Hayward et al., 2002; Rodriguez et al., 2002; Rodriguez et al., 2005), inhibit metal coordination (Rodriguez et al., 2005) and/or reduce the repulsive negative charge (Sandelin et al., 2007).

The aim of this study is to investigate if and how T18S and E40K mutations promote protein aggregation of canine SOD1. These two mutations (corresponding to c.52A > T and c.118G > A mutations in the canine SOD1 gene) are unique to canine degenerative myelopathy (DM), a neurodegenerative disease in dogs clinically and histologically similar to ALS (Awano et al., 2009). In a homology model of canine SOD1 (Figs. 1A and 1B), the T18 residue (corresponding to the I17 residue in human) is located in the dimer interface and forms a hydrophobic contact with the T52 residue of the other subunit. The E40 residue is exposed to the solvent and forms a salt bridge with the K91 residue of the same subunit. Since we previously reported that the T18S and E40K mutations promote formation of insoluble aggregates in neurons and glial cells (Kobatake et al., 2017), here we studied the pathological consequences of these two mutations at the level of protein folding. We also investigated the molecular mechanism underlying canine SOD1 aggregation.

## Materials & Methods

### Recombinant canine SOD1 expression and purification

Bacterial expression plasmid vectors, pGEX4T-1 (GE Healthcare Bio-Sciences AB), for the production of canine recombinant SOD1 wild-type (WT) and E40K proteins were generated as described previously (Nakamae et al., 2015). The plasmid harboring the T18S mutation was obtained by site-directed mutagenesis of the SOD1 WT plasmid using the NEBuilder HiFi DNA Assembly Cloning Kit (New England Biolabs Inc). The primer pair used was 5′-GTGGAAGGCTCCATTCATTTTGTGCAGAAA-3′ and 5′-TTTCTGCACAAAATGAATGGAGCCTTCCAC-3′. The presence of the T18S mutation was confirmed by DNA sequence analysis. The pGEX4T-1 expression plasmids were transformed into competent (BL21) *E.coli* cells (Thermo Fisher Scientific) and plated on LB agar with 100 µg/ml ampicillin at 37 °C overnight. Single colonies were inoculated into LB media with 100 µg/ml ampicillin and grown at 37 °C overnight, then diluted 1:20 in fresh LB media with 100 µg/ml ampicillin and grown at 37 °C for 2 h. Protein expression was induced by addition of 1.0 mM isopropyl *β*-D-1-thiogalactopyranoside (IPTG) at 20 °C for 20 h. Cell suspensions were centrifuged at 3,500 × g for 20 min, washed with 1 × PBS and centrifuged at 14,000 × g for 20 min. Cell pellets were stored at −30 °C. The cell pellets were suspended in lysis buffer (10 mM Tris, 1 mM EDTA and 150 mM NaCl) with 200 µg/mL lysozyme and 200 µg/mL phenylmethylsulfonyl fluoride (PMSF), then disrupted by sonication on ice and centrifuged at 14,000 × g for 20 min. The supernatant was filtered by 0.45 µm FILSTAR Syringe Filter (Hawach Scientific) and applied onto a Glutathione Sepharose 4 FF column (GE healthcare). The bound SOD1 proteins were cleaved on the Glutathione Sepharose by addition of 400 U thrombin (FUJIFILM Wako Pure Chemical Corporation) at 22 °C overnight. After removing thrombin using a HiTrap Benzamidine FF column (GE Healthcare), the Cu^2+^ and Zn^2+^-free SOD1 (apo-SOD1) were generated by incubation in 40 mM sodium phosphate (pH 7.4) containing 10 mM EDTA and concentrated by Amicon Ultra Centrifugal filters with a 10 K membrane (Millipore). The Cu^2+^ and Zn^2+^-bound SOD1 (holo-SOD1) were generated using the procedure previously described by Crisp et al. (2013) with some modification. Briefly, the apo-SOD1 was dialyzed with 100 mM Tris (pH8.0), 300 mM NaCl and 200 µM CuCl_2_ for 3.5 h, and then with 100 mM Tris (pH8.0), 300 mM Tris and 200 µM ZnSO_4_ for 3.5 h. The holo-SOD1 was dialyzed with 20 mM Tris and 10 mM NaCl overnight at 4 °C, and then concentrated using the Amicon Ultra Centrifugal filters. Protein concentrations were determined with the absorption at 280 nm using a UV spectrometer (Shimadzu).

The secondary structure of each protein was examined by circular dichroism (CD). The CD spectra were acquired using a Chirascan spectrophotometer (Applied Photophysics). Net charges of the SOD1 proteins were calculated using a protein analysis software (PROTEIN CALCULATOR v3.4).

### SOD1 activity assay

The SOD1 activity was determined using SOD Assay Kit-WST (DOJINDO LABORATORIES) according to a protocol provided by the manufacture. Each SOD1 protein was diluted to 250 µg/mL with 40 mM sodium phosphate (pH 7.4). The assay was repeated three times.

### Thioflavin T assay

For amyloid fibril formation, solutions containing 40 µM SOD1 and 10 µM thioflavin T (ThT) were shaken at a rate of 170 rpm in a 96-well plate at 37 °C. For the experiments using apo-SOD1, 10 mM EDTA was added to the above solutions. Amyloid fibril assembly was monitored by the change in ThT fluorescence at 495–505 nm (excitation wavelength of 405 nm) using GloMax Discover Microplate Reader (PromegaKK).

### Transmission electron microscopy

Samples containing SOD1 proteins were adsorbed onto carbon-coated 200-mesh copper grids (EM Japan Co., Ltd.) and negatively stained with 2% w/v phosphotungstic acid. Transmission electron microscopy (TEM) was performed using JEM-2100F operating at 200 kV (JEOL Ltd.).

### Structural stability of SOD1

The structural stability of SOD1 was examined by monitoring thermal denaturation using CD. Far-UV CD spectra were acquired in the wavelength range of 195–260 nm using a 1 mm pathlength cuvette. Thermal denaturation was examined by scanning the temperature from 20 to 95 °C at 1 °C/min and monitoring *θ*_220_. The SOD1 proteins were diluted to 10 µg/mL with 40 mM sodium phosphate (pH 7.4).

The GdnHCl-induced unfolding was examined by monitoring the CD. The SOD1 proteins were diluted to 110 µg/mL with 5 mM HEPES (pH 7.4) containing various concentrations of GdnHCl. The solutions were incubated at 37 °C for 1 h and then CD values at 220 nm were recorded. Each examination was performed three times individually.

### Statistical analysis

Multiple group means were compared by one-way ANOVA with Tukey’s multiple comparison tests for pair-wise comparisons.

## Results

### Production of canine holo- and apo-SOD1

**Figure 2:**
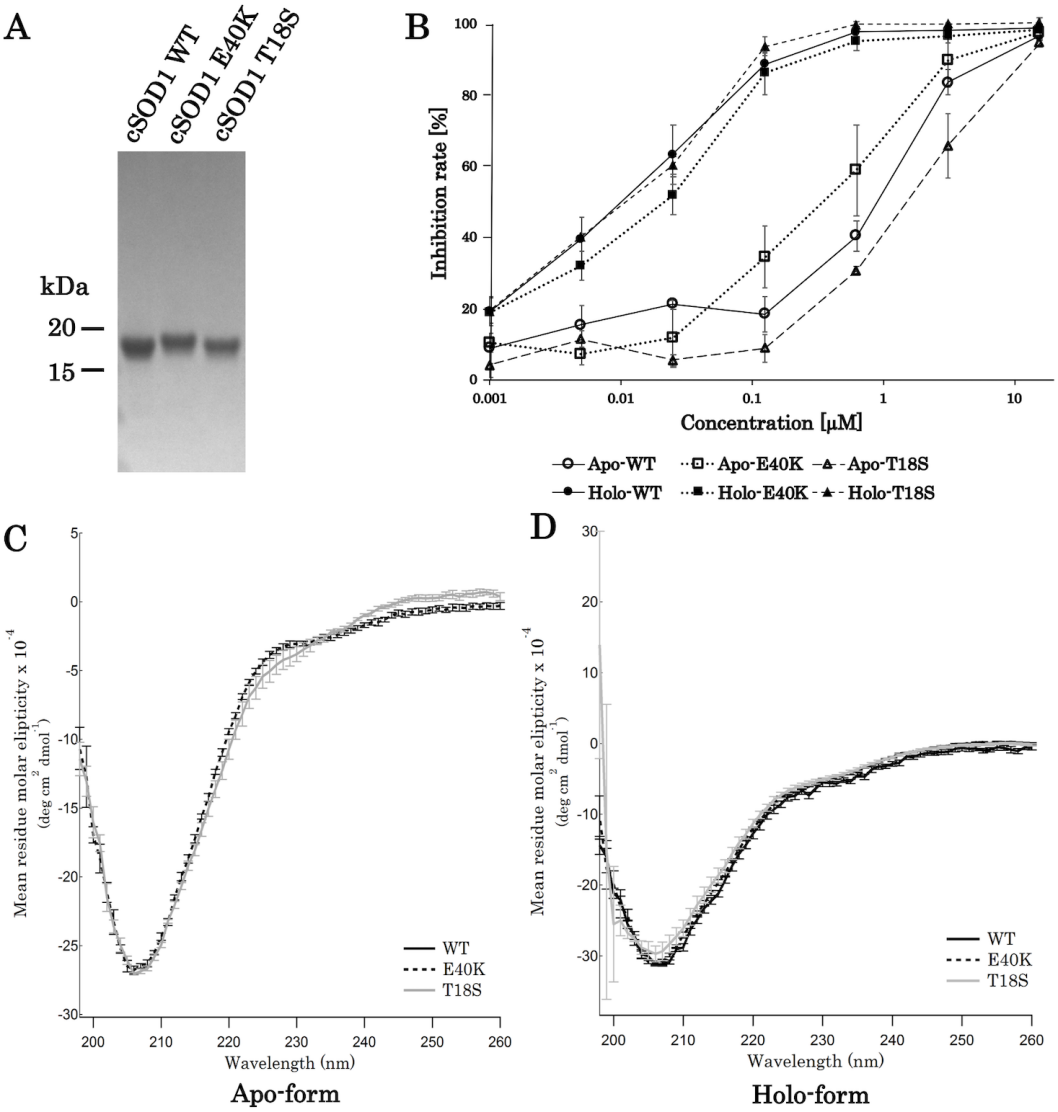
Expression and purification of canine SOD1. (A) A Coomassie stained SDS-PAGE gel of purified canine SOD1. (B) Enzymatic activity of canine SOD1. The activities of Cu^2+^/Zn^2+^-bound canine SOD1 (holo-) were significantly higher than those of Cu^2+^/Zn^2+^-unbound canine SOD1 (apo-). No significant difference was found between WT, E40K and T18S canine SOD1. Each data point is mean ± SEM. (C and D) Far-UV circular dichroism spectra of (C) apo- and (D) holo-WT, E40K, and T18S canine SOD1. Each data point is mean ± SEM.

For the production of canine SOD1 proteins, we used a bacterial expression system and a one-step affinity-chromatography. SDS-PAGE of the purified SOD1 demonstrated a single homogeneous band at 16 kDa (Fig. 2A and Fig. S1), consistent with the molecular size predicted from the amino acid sequence. We produced Cu^2+^/Zn^2+^-unbound SOD1 (“apo-SOD1”) and -bound SOD1 (“holo-SOD1”) using a dialysis procedure described in the Materials & Methods section. As show in Fig. 2B and Data S1, enzymatic activity of holo-SOD1 was ∼100-fold higher than that of apo-SOD1, suggesting that the major population of apo-SOD1 (∼99%) was deprived of Cu^2+^/Zn^2+^ by the procedure.

A far-UV CD spectra of holo-SOD1 represented a negative minimum at 208 nm (Fig. 2D), indicating the formation of an anti-parallel *β*-sheet structure (Higurashi et al., 1999). No difference in the CD spectra was found between holo-SOD1 and apo-SOD1, consistent with previous reports in human SOD1 (Benatar, 2007). Overall, the structural and enzymatic characteristics of canine SOD1 were in good agreement with those of human SOD1.

### The E40K and T18S mutations did not affect global fold of SOD1

Next, we produced canine SOD1 harboring the E40K or T18S mutation. No significant difference in enzymatic activity or CD spectrum were found between WT and the mutant SOD1 (Figs. 2B–2D), indicating that the E40K and T18S mutations did not significantly affect the global fold of canine SOD1.

### The E40K and T18S mutations promote the production of worm-like fibrils

**Figure 3:**
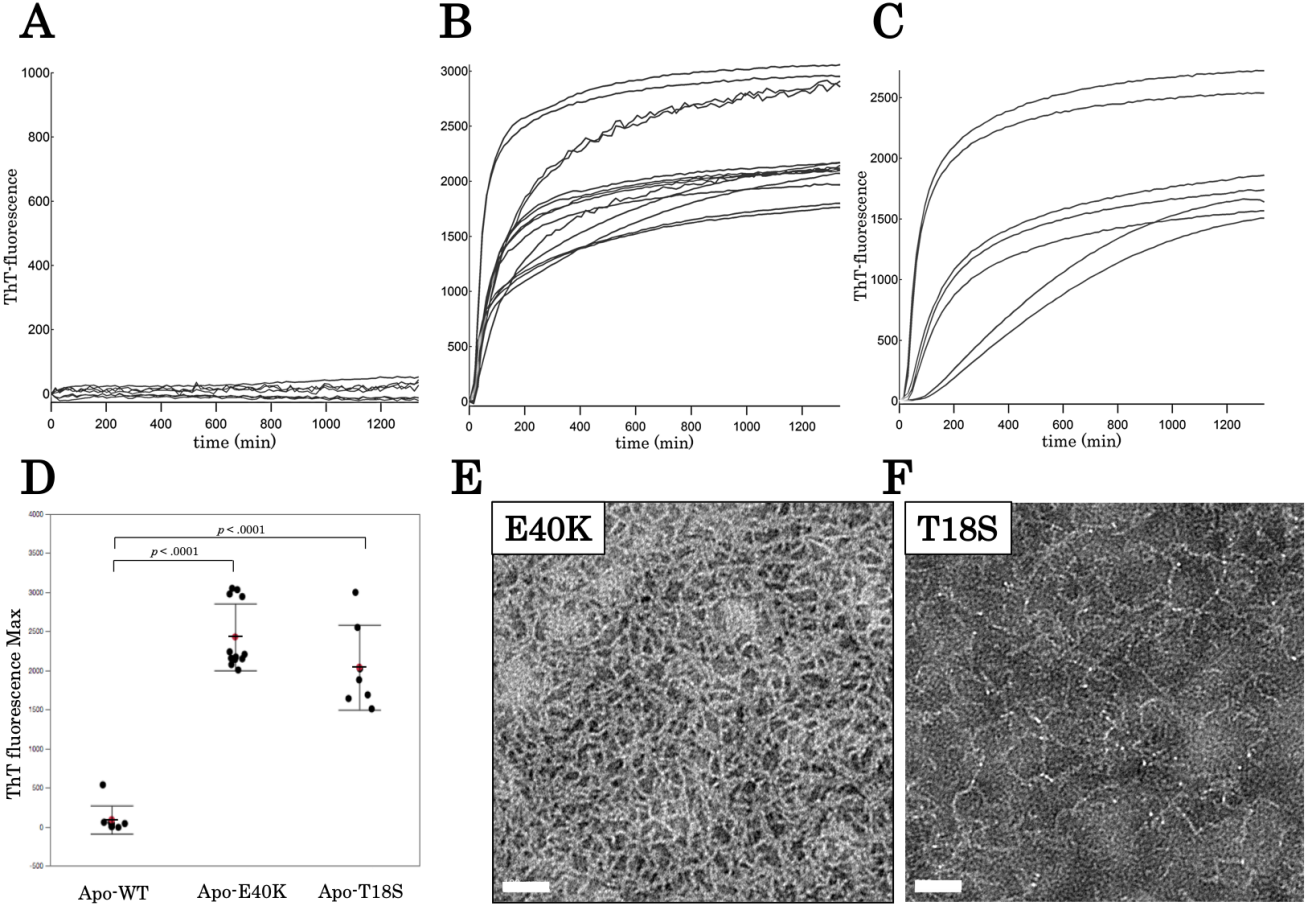
Time dependent thioflavin T assay and electron micrographs of canine SOD1 fibrils. (A–C) Time courses of aggregation of WT, E40K, and T18S canine SOD1 in the Cu^2+^/Zn^2+^—unbound state. (D) The maximum of ThT fluorescence derived from Figs. 3A–3C. Both the E40K and T18S mutations significantly increased the ThT fluorescence (*p* < 0.001). (E and F) Negatively stained electron microscopy images of SOD1 E40K (E) and SOD1 T18S (F). Thin, curved, and fibrous aggregates were observed. Scale bar = 100 nm.

To characterize aggregation properties of the canine SOD1, we performed a Thioflavin-T (ThT) assay. The holo-SOD1 did not give rise to a ThT-positive aggregate, even in the presence of mutations (Data S2). Similarly, apo-SOD1 without mutation did not give rise to a ThT-positive aggregate (Figs. 3A and 3D). However, apo-SOD1 harboring E40K or T18S mutation produced ThT-positive aggregates after 0–200 min shaking (Figs. 3B–3D).

We examined morphology of the ThT-positive aggregates using negatively stained electron microscopy. As shown in Figs. 3E and 3F, we observed curvy fibrous aggregates, typically termed “worm-like fibrils” (Singh et al., 2012), in the E40K and T18S SOD1 aggregates (Figs. 3E and 3F). By contrast, we did not observe any aggregates in WT apo-SOD1 or holo-SOD1 (Fig. S2). Thus, the E40K and T18S mutations promote formation of ThT-positive amyloid-like aggregates.

### The T18S mutation and the E40K mutation did not reduce the global structural stability

**Figure 4:**
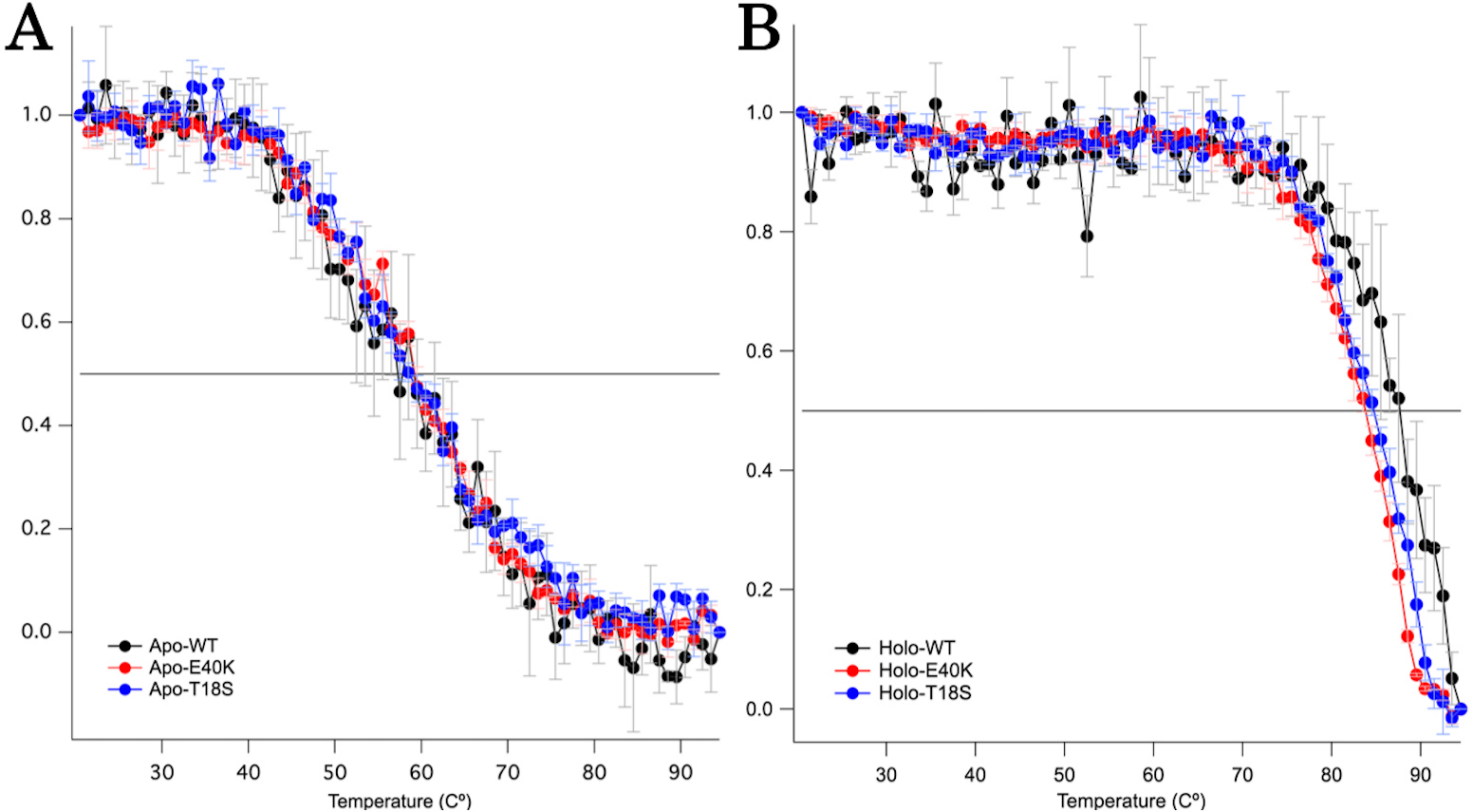
Thermal denaturation curves of (A) apo- and (B) holo- canine SOD1. The vertical axis shows a normalized CD value at 220 nm. Each data point is mean ± SEM.

**Figure 5:**
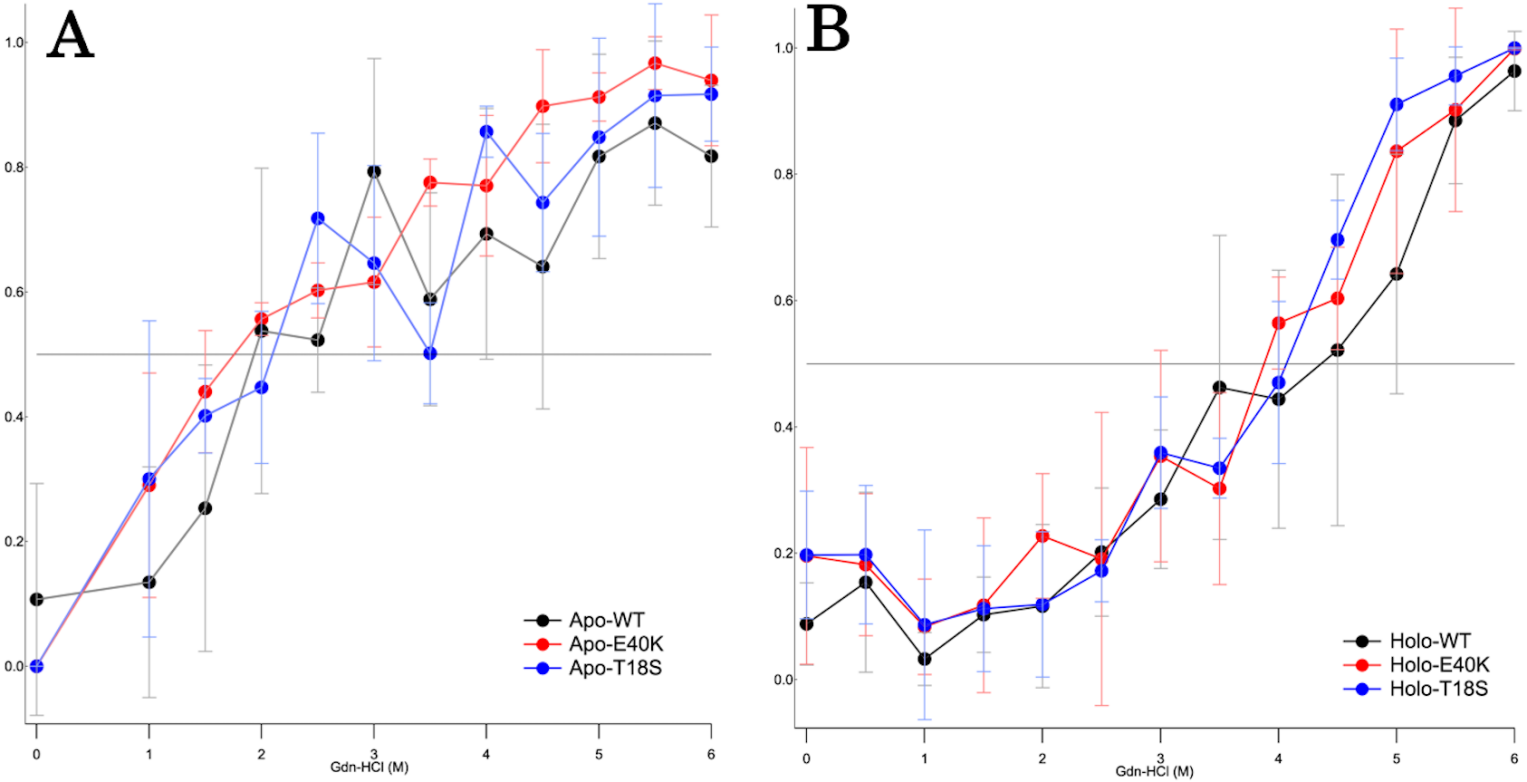
The GdnHCl-denaturation curves of (A) apo- and (B) holo-canine SOD1. The vertical axis shows the fraction of unfolded protein (*f*_*D*_) derived from CD values at 220 nm. Each data point is mean ± SEM.

To characterize the mechanism of aggregation, structural stabilities of SOD1 were examined using thermal and GdnHCl denaturation assays and far-UV CD. Interestingly, thermal denaturation curves of WT, E40K, and T18S SOD1 were almost superimposable, both in the apo- and holo-SOD1 (Fig. 4 and Data S3). Furthermore, GdnHCl denaturation curves of the three SOD1 were also superimposable (Fig. 5 and Data S4). Thus, the T18S and the E40K mutation did not reduce the structural stability of SOD1, at least at the level of secondary structure.

## Discussion

Understanding the mechanism of protein aggregation is crucial to elucidate ALS pathology. Although a number of studies have been performed on the human SOD1 (Shaw & Valentine, 2007), there are a few reports regarding canine SOD1 (Crisp et al., 2013). Moreover, several mutations in canine SOD1 have been linked to DM, but whose phenotype is uncharacterized at the level of protein folding. Thus, it is important to understand how the canine SOD1 mutations would alter protein folding of SOD1. It is also valuable to compare the mechanisms of protein aggregation between canine and human SOD1 to establish the pathological analogy between DM and ALS. Here, we investigated mechanism of canine SOD1 aggregation induced by the E40K and T18S mutations.

Regardless of the absence or presence of the mutations, canine SOD1 can adopt both holo- and apo-states (Fig. 2B), consistent with human SOD1. The structural stability of apo-SOD1 was significantly lower than that of holo-SOD1 (Figs. 4 and 5) (Furukawa & O’Halloran, 2005). Typically, protein aggregation begins with the formation of unstable intermediates. Thus, the unstable apo-SOD1 might be the initial intermediate in canine SOD1 aggregation. Indeed, we found that apo-SOD1, but not holo-SOD1, gave rise to fibrous aggregates in the presence of the pathogenic mutations (Figs. 3A–3D).

The canine SOD1 harboring the T18S mutation had a high propensity to aggregate (Fig. 3C). Interestingly, the T18S mutation did not reduce the structural stability against unfolding, at least at the level of secondary structure (Fig. 4). We postulate that the dimer interaction between the T18 and T54 residues might be lost by the T18S mutation (Fig. 1B). Alternatively, the T18S mutation might rearrange the local structure around *β*2, such that the C6 residue is exposed to the solvent. In human SOD1, previous evidence suggests that free Cys residues attack the disulfide bond between C57 and C146 residues and produce disulfide-linked SOD1 oligomers (Toichi, Yamanaka & Furukawa, 2013). Furthermore, exposure of C6 was suggested to trigger the formation of disulfide-linked oligomers (Niwa et al., 2007). Thus, the T18S mutation might promote protein aggregation, through the production of disulfide-linked oligomers.

The canine SOD1 harboring the E40K mutation also promoted protein aggregation without reducing the global structural stability (Figs. 4 and 5). The canine SOD1 has a negative net charge (−4.5) at a physiological pH and the E40K mutation decreases the negative net charge to −2.0 (Table 1). A decreased net charge promotes protein aggregation by enhancing intermolecular electrostatic forces between unfolded polypeptides (Chiti et al., 2002; Calamai et al., 2003). In fact, the D125H, D90A, E100K, D101N or N139K mutations in human SOD1 were suggested to promote protein aggregation by decreasing the repulsive negative charge (Shaw & Valentine, 2007). The same mechanism might be applied to the E40K mutation. Alternatively, the E40K mutation might promote protein aggregation, through disrupting the salt bridge between the E40 and K91 residues (Fig. 1B). Several pathogenic mutations in human ALS are found in the region surrounding the E40-K91 salt bridge (including G93A and H43R), suggesting the importance of the salt bridge in disease prevention. Consistently, a recent computer-based study has suggested that the E40-K91 salt bridge is crucial for stabilizing the *β*-barrel core and Zn-binding domain of SOD1 (Crown et al., 2019).

**Table 1:** Net negative charge of canine SOD1 (pH 7.4).

**Protein**	**Charge**
WT	−4.5
E40K	−2.5
T18S	−4.5

The morphology of the fibrous aggregates observed in our study was different from typical amyloid fibrils. Typical amyloid fibrils are composed of bundled straight and rigid fibrils, but canine SOD1 fibrils were composed of unbundled curvy fibrils (Figs. 3E and 3F). Moreover, the kinetics of canine SOD1 aggregation did not show a lag phase (Fig. 3B and 3C), which was inconsistent with the typical amyloid fibrils. Thus, a further study is needed to determine whether the canine SOD1 aggregates are *bona fide* amyloid fibrils and involved in the DM pathogenesis.

## Conclusions

In summary, our study revealed that canine SOD1 harboring T18S or E40K mutations promote protein aggregation without reducing the global structural stability. Further study using high resolution techniques, such as NMR, is required to investigate whether these mutations rearrange the local structure, reduce the intermolecular repulsive forces, and promote disulfide-linked oligomers.

##  Supplemental Information

10.7717/peerj.9512/supp-1 Data S1 SOD activity raw data

10.7717/peerj.9512/supp-2 Data S2 Aggregation kinetics raw data

10.7717/peerj.9512/supp-3 Data S3 *Tm* CD raw data

10.7717/peerj.9512/supp-4 Supplemental Information 4 *C*_*m*_ CD raw data

10.7717/peerj.9512/supp-5 Figure S1 A Coomassie stained SDS-PAGE gel of purified canine SOD1

10.7717/peerj.9512/supp-6 Figure S2 Negatively stained electron microscopy images of canine SOD1 No aggregates were observed in apo-SOD1 WT (A), holo-SOD1 WT (B), E40K (C) and T18S (D). Scale bar = 100 nm.

## Additional Information and Declarations

### DNA Deposition

The following information was supplied regarding the deposition of DNA sequences: The canine SOD1 sequences are available at GenBank: NM_001003035.1. The 3D structure of human SOD1 protein are available at Protein Data Bank: 3ECU.
https://www.rcsb.org/structure/3ECU.

### Data Availability

The following information was supplied regarding data availability: The raw measurements are available in the Supplemental Files.
