# Supplementary figures and images for: Canine SOD1 harboring E40K or T18S mutations promotes protein aggregation without reducing the global structural stability
Source: PeerJ. 2020 Jul 15;8:e9512. doi: 10.7717/peerj.9512 (PMC7368427; doi:10.7717/peerj.9512)

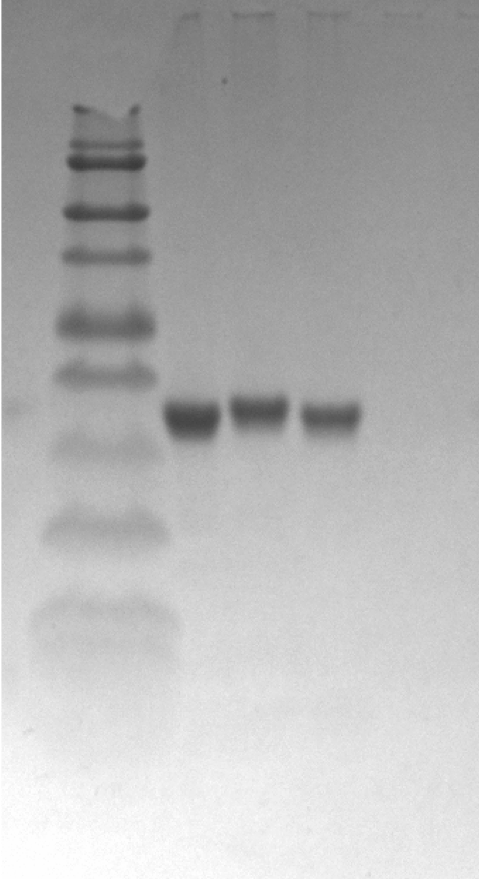

Supplement: Figure S1 [file peerj-08-9512-s005.png]

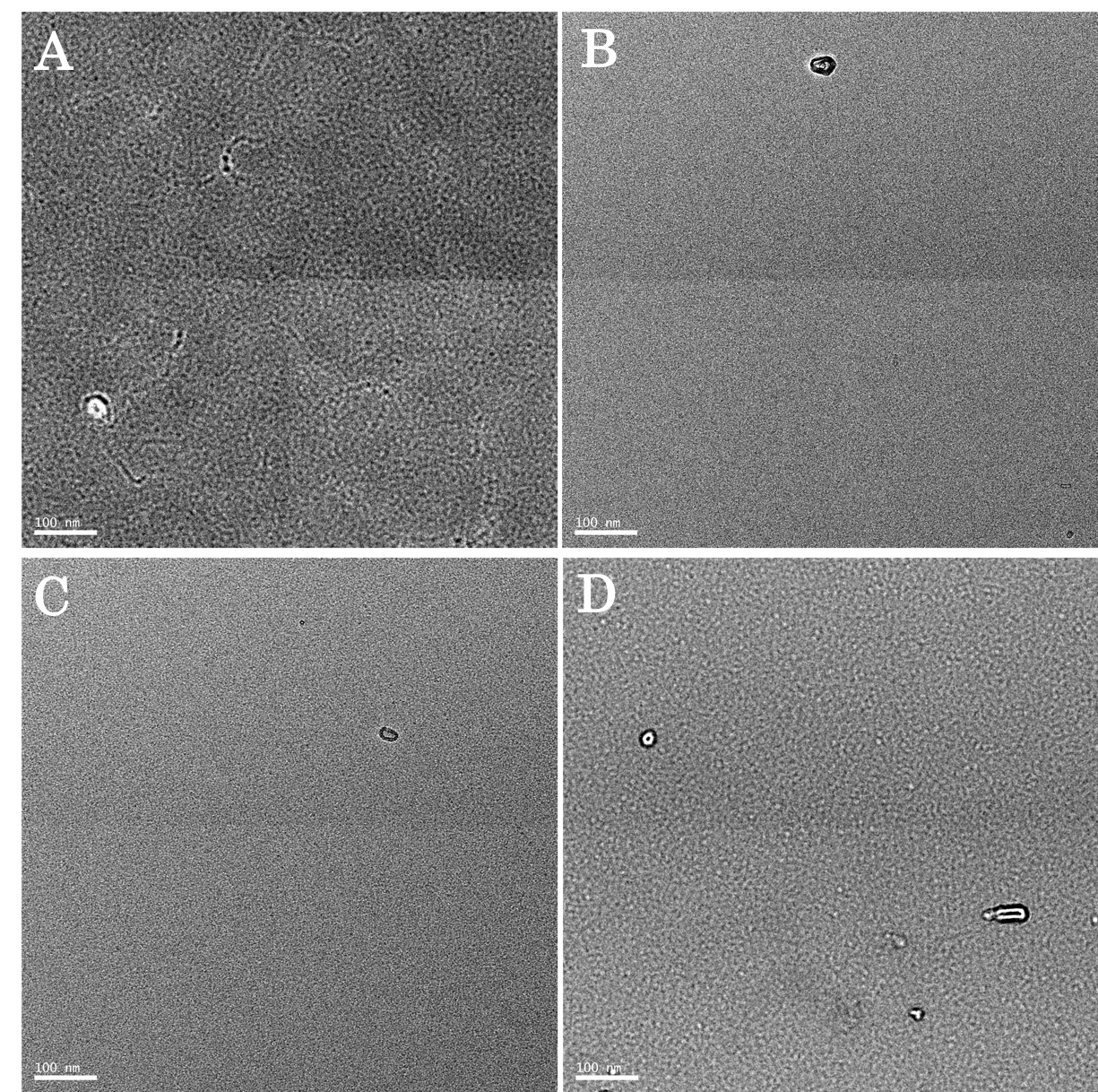

Supplement: Figure S2 — No aggregates were observed in apo-SOD1 WT (A), holo-SOD1 WT (B), E40K (C) and T18S (D). Scale bar = 100 nm. [file peerj-08-9512-s006.png]
